# Urinary neopterin and total neopterin measurements allow monitoring of oxidative stress and inflammation levels of knee and hip arthroplasty patients

**DOI:** 10.1371/journal.pone.0256072

**Published:** 2021-08-17

**Authors:** Gregory Baxter-Parker, Lloyd Roffe, Elena Moltchanova, Jay Jefferies, Siddarth Raajasekar, Gary Hooper, Steven P. Gieseg

**Affiliations:** 1 School of Biological Sciences, University of Canterbury, Christchurch, New Zealand; 2 Department of Orthopaedic Surgery & Musculoskeletal Medicine, University of Otago Christchurch, Christchurch, New Zealand; 3 School of Mathematics and Statistics, University of Canterbury, Christchurch, New Zealand; 4 Department of Radiology, University of Otago Christchurch, Christchurch, New Zealand; University of Messina, ITALY

## Abstract

Knee and hip arthroplasty are common surgeries within an aging population. Some data has suggested that knee arthroplasty is more traumatic to the body than hip arthroplasty due to the increased complexity and load bearing nature of the joint. Here, we compare the stress of the two surgeries by measuring urinary neopterin and total neopterin as biomarkers of oxidative stress and inflammation. Urinary neopterin and total neopterin (neopterin + 7,8-dihydroneopterin) levels were analysed in 28 knee and 22 hip arthroplasty patients pre- and post-operatively to determine oxidative stress and inflammation levels. Total neopterin was 31.1% higher with knee arthroplasty (p<0.05). Urinary neopterin was 32.8% higher in the knee arthroplasty group versus hips. The increase in neopterin and total neopterin following a post-surgical decrease in levels was significant in both groups. Levels of neopterin and total neopterin were varied between patients, but all increased following surgery and subsided by day 28. The increased levels of urinary neopterin and total neopterin from knee arthroplasty indicate that knee osteoarthritis and arthroplasty is a more significant trauma to the body than hip osteoarthritis and arthroplasty surgery. This is also shown by faster inflammatory resolution following hip arthroplasty.

## Introduction

Surgery is deliberate but controlled trauma to the body and as a result, sets off a range of stress responses including inflammatory cascades. The levels of tissue disruption from the surgical trauma is expected to be reflected in the scale of the body’s response especially in the level of inflammation. Hip arthroplasty is often reported by surgeons to be less traumatic to the body than knee arthroplasty, which is reflected in poorer functional outcomes after 5 years and decreased reported quality of life [1,2]. It has been suggested that the difference in outcome is because the knee is a more complicated joint that also has to balance off-center loads, thus making it is more susceptible to complications.

The biochemically measurable difference in physical trauma and inflammation between hip and knee replacement surgery is subtle, with one study showing increased levels of hormonal markers (noradrenaline, adrenaline and glucose) in knee arthroplasty versus hip, but not inflammatory markers interleukin-6 (IL-6) or C-reactive protein (CRP) [3]. While IL-6 and CRP are commonly used biomarkers of immune system activity and inflammation, they could not be used to distinguish between knee and hip arthroplasty surgeries in multiple studies [3–5]. Additionally, these two biomarkers return to pre-surgical levels within one week, in spite of a physically observable (redness, swelling and bruising) level of inflammation around the wound [6]. The inability of IL-6 or CRP to quantify post-surgical inflammation may be due to the level of biochemical inflammation not being reflected in the visual signs, or alternatively, these biomarkers may lack the sensitivity required to show subtle differences in immune response. Another limitation of these markers is they require the collection of blood, an additional invasive post-surgical procedure for patients.

Neopterin and its unoxidized form 7,8-dihydroneopterin, are relatively sensitive markers of inflammation as they are generated within the site of inflammation. 7,8-Dihydroneopterin is a monocyte/macrophage synthesized antioxidant which is produced upon interferon-gamma stimulation. 7,8-Dihydroneopterin rapidly scavenges superoxide and hypochlorite, both products of the inflammatory response, to generate the highly fluorescent neopterin [7–11]. These factors allow the duality of inflammation and oxidative stress measurement using 7,8-dihydroneopterin and neopterin [11–15]. Neopterin can be easily detected in urine due to its highly fluorescent characteristics, however, 7,8-dihydroneopterin does not share neopterin’s highly fluorescent properties and must be oxidized into neopterin by acid iodide treatment for detection by fluorescence spectroscopy. This combination of neopterin and 7,8-dihydroneopterin is called “total neopterin” [16].

The ratio of total neopterin to neopterin can be used to investigate the environmental dynamics between monocyte/macrophage activation and oxidative stress. A low ratio signifies an environment where there is a lot of oxidative stress, relative to immune activation, whereas a high ratio indicates an environment in which there is a high level of immune cell activation with relatively less oxidative stress [14,17,18]. This measure must however be taken into consideration with the raw neopterin and total neopterin values as the ratio alone is not indicative of oxidative stress or immune cell activation levels.

Neopterin and 7,8-dihydroneopterin is rapidly cleared by the kidneys and concentrated in the urine thus urinary neopterin appears to provide a clearer report of inflammation than blood analysis when corrected for urine concentration [19–22]. As a result, urinary neopterin has been extensively used as a marker of inflammation which also avoids the need for invasive blood collection [14,23,24].

Total neopterin (neopterin + 7,8-dihydroneopterin) and neopterin have been used as sensitive biomarkers of inflammation and oxidative stress respectively. They have been used extensively to monitor immune system activation in illness and trauma, allowing for quantitative analysis of patient’s inflammatory recovery [12,17,25]. Analysis of these biomarkers has shown that they can be used to monitor individual responses to exercise induced trauma, allowing for tailored training regimes to suit individuals. In a previous study, neopterin and total neopterin indicated elevated levels of oxidative stress and inflammation following knee arthroplasty surgery versus healthy controls [25].

In this study we have used urinary neopterin and total neopterin to assess differences in oxidative stress and inflammation levels between knee and hip arthroscopy patients over 28 post-operative days as well as assessing the inflammatory recovery for each type of surgery. We hypothesized that firstly, knee arthroplasty would yield higher values for urinary neopterin and total neopterin than hip arthroplasty, and secondly, hip arthroplasty would demonstrate a more rapid reduction in biomarker levels.

## Materials and methods

### Study design and population

This was an observational cohort study investigating a diagnostic test with Level III evidence. The surgical cohort comprised of knee and hip arthroplasty patients. Knee arthroplasty patients (n = 28) were aged 68.00 ± 10.37 years (mean ± SD) and 54% were female. The hip arthroplasty patients (n = 22) were aged 66.26 ± 7.77 years (mean ± SD) and 52% female. Ethics approval (New Zealand Health and Disability Ethics Committee 16CEN84AM02) and informed consent was gained from the surgical patients. Exclusion criteria were; patients below 18 or above 80 years of age, smokers, or patients having recently received a diagnosis of cancer. The effect of sex or age between the exclusion criteria range has been shown to not affect neopterin levels. Patients provided urine samples before surgery, immediately after surgery, and each day following at approximately midday (± 2 hours) until the patients were discharged. Day 14 and 28 day samples were collected from the patient’s home when possible.

### Surgery description

For knee arthroplasty, all patients had a tourniquet applied proximal to the surgical site and inflated to 350 mmHg. An anterior midline incision was made over the knee with a medial parapatellar dissection and exposure of the joint. Jig-assisted preparation of the distal femur and tibial plateau was performed with subsequent insertion of the appropriate implants. All components were uncemented in each patient and all wounds were drained for 24 hours. For hip arthroplasty patients the hip was dislocated and the femoral head removed with a power saw. The acetabulum was prepared with power reamers and sized to accept an uncemented shell and a highly cross linked polyethylene liner. No image guidance was used. The femur was reamed by hand and the appropriate uncemented or cemented stem inserted following trial reduction. Leg lengths and off-set were measured and the appropriate femoral ceramic head inserted. Following final reduction, the wound was closed in layers over a pain catheter. No drains were used. All patients for both surgeries received intravenous Tranexamic Acid (1g) following the procedure to reduce blood loss.

### Anaesthetic description

All anaesthetics were performed as per each anaesthetist’s routine practice. Standard medications used to induce and maintain anaesthesia for all patients were Midazolam, Fentanyl, Propofol, Dexamethasone, Rocuronium, Ondansetron, and Cyclizine. At induction all patients were given intravenous Cefazolin (2g) which was continued for a further 24 hours post-operatively. Post-operative pain management was obtained using Paracetamol, Codeine and Fentanyl.

### Sample preparation and specific gravity measurements

Urinary samples were provided by patients into 70 mL sample bottles in the orthopaedic recovery wards and immediately refrigerated in the absence of light. Samples were taken pre-surgery, as soon as the patient was able post-surgery, and then at 12 pm on each following day until the patient was discharged (typically 3–4 days). The samples were collected daily and transported on ice to the University of Canterbury before being aliquoted and stored in a -80°C freezer.

Urine samples were thawed in the dark to remove any ultraviolet light mediated oxidation of 7,8-dihydroneopterin. 5 μL of urine was mixed with 195 μL of 20 mM ammonium phosphate (pH 2.5), with 100 μL transferred into high-performance liquid chromatography (HPLC) vials for neopterin determination. Of the remaining 100 μL of diluted sample, 20 μL of acidic iodide was added, followed by mixing on a vortex mixer and a 15 minute incubation in the absence of light at room temperature to oxidise the 7,8-dihydroneopterin into neopterin for total neopterin analysis. The unreacted acidic iodide was neutralised by addition of 10 μL of 0.6 M ascorbic acid followed by further mixing using a vortex mixer. 100 μL of this solution was transferred into HPLC vials for total neopterin determination.

HPLC measurements were made using a Shimadzu 20A HPLC with a Sil-20A autosampler and RF-20Axls fluorescence and M20A SPD absorbance detectors. Separation of neopterin was achieved using a Phenomenex Luna 5 μm Strong Cation Exchange 100 Å 250 mm × 4.6 mm column. The mobile phase consisted of 20 mM ammonium phosphate at pH 2.5 being pumped at 1.0 ml min-^1^. Fluorescence wavelengths for neopterin detection were set at 438 nm and 353 nm for emission and excitation, respectively. Peak analysis and result quantification was conducted using Shimadzu Lab Solutions version 5.86.

Neopterin and total neopterin values were standardised using specific gravity (SG) SG was measured using an ATAGO N-20 refractometer, and calculated with the formula described below, based on the normal population SG_1.020_ [21]. Samples were bought to room temperature to remove and temperature-dependent density variation bias.

[neopterin](nM/SG_1.020_) = (SG_1.020_−1) / (SG_sample_ -1) × [neopterin](nM)

### Geological information

The present study was conducted in Christchurch, New Zealand. All patients from the surgical cohort were operated on and cared for in Burwood Hospital, Christchurch, New Zealand. Day 14 and 28 post-surgical samples were, when required, collected from patients homes around Christchurch and Canterbury, New Zealand.

### Statistical analysis

A repeated measures 2-way ANOVA was performed to test the effects of surgery type and time of measurement on the biomarkers levels. The response variables were logged to improve the normality and heteroscedasticity of the residuals and of random effects, and the models were checked for these diagnostics. All modeling was done using the R-packages lme4 and lmerTest [26,27].

## Results

**Fig 1:**
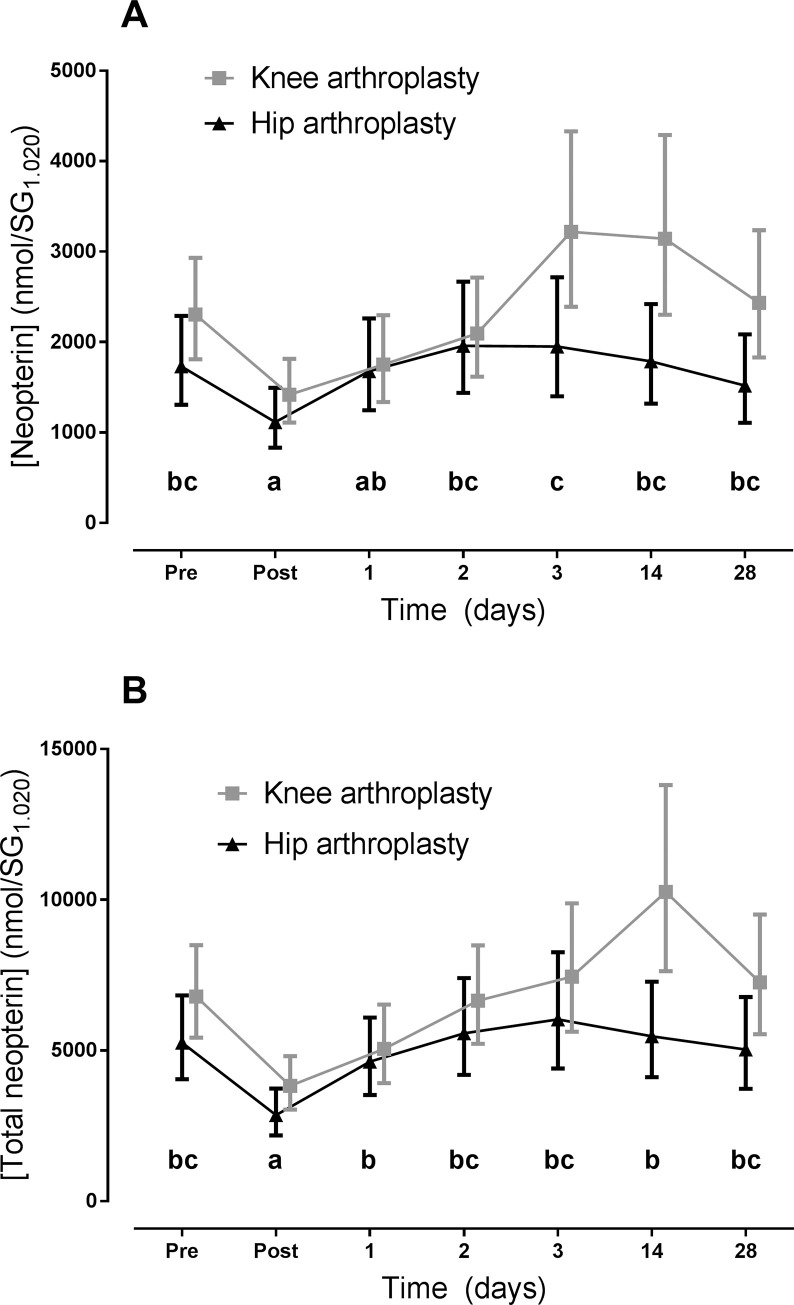
Urinary neopterin (A) and total neopterin (B) mean estimated levels and 95% confidence intervals for knee and hip arthroplasty patients before and after surgery. Urinary neopterin and total neopterin levels are on average higher for knee arthroplasty by 32.8% (ns) and 31.1% (p<0.05), respectively. There was a significant reduction in neopterin and total neopterin (p<0.001) for both groups between pre- and post-surgical samples, with most additional daily values being significantly higher than the post-surgical level in both groups (Tukey letter plots). No statistically significant differences were found for time instances sharing a letter (Tukey post-hoc pairwise comparisons test).

Urinary neopterin and total neopterin were measured to determine the levels of oxidative stress and inflammation in knee and hip arthroplasty patients. Pre-surgical urinary neopterin and total neopterin levels were higher in knee arthroplasty patients than hip patients. Post-surgical urinary neopterin and total neopterin levels dropped significantly (p<0.01) from pre-surgical values for both groups. From day 2 onwards, urinary neopterin and total neopterin were elevated significantly from the post-surgical measurement in both groups, but not the pre-surgical value. Total neopterin was on average 31.1% higher for knee arthroplasty than hip (p<0.05) over all days (Fig 1B), indicating a significantly higher level of inflammation in the knee arthroplasty group. The group data urinary neopterin was, on average over all days, 32.8% higher with knee arthroplasty than hip arthroplasty (Fig 1A), but the difference was not statistically significant (p = 0.06). While the average difference in biomarker levels over every time point is significant, there were no statistically significant differences between knee and hip arthroplasty for any individual day. However, mean urinary neopterin and total neopterin trend was considerably higher for knee arthroplasty at day 14 post-surgery, although the difference was not statistically significant.

**Fig 2:**
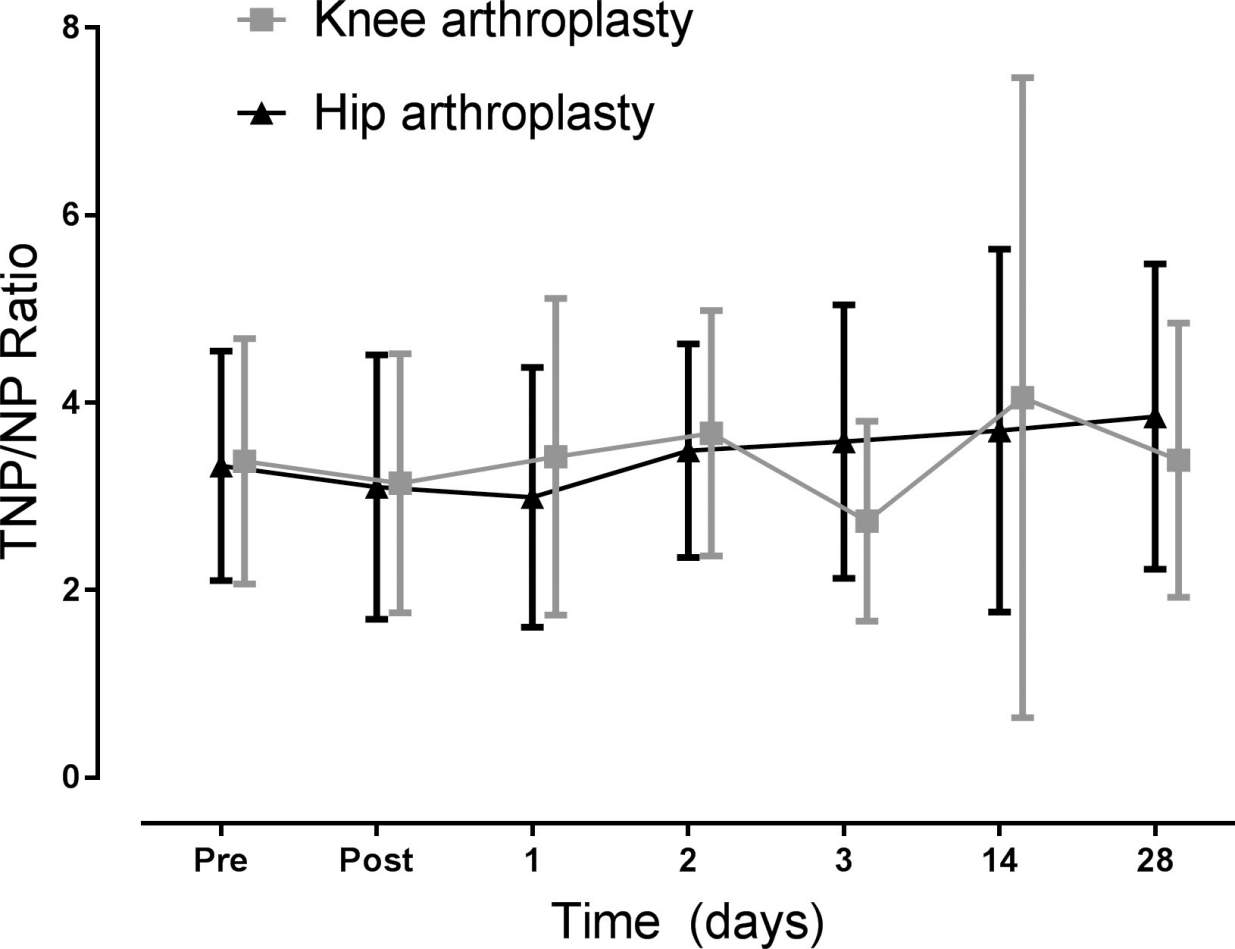
Total neopterin over neopterin ratios for knee and hip arthroplasty patients before and after surgery. There are no statistically significant differences in the TNP/NP ratios between the groups at any day. Data is presented as means with standard deviation.

The TNP/NP ratio gives an indication of inflammation versus oxidative stress levels [17]. In this study, there were no differences in TNP/NP ratio between knee and hip arthroplasty surgeries at any day (Fig 2). Peak mean TNP/NP ratios were 4.06 for knee arthroplasty on day 14 and 3.85 for hip arthroplasty at day 28.

**Fig 3:**
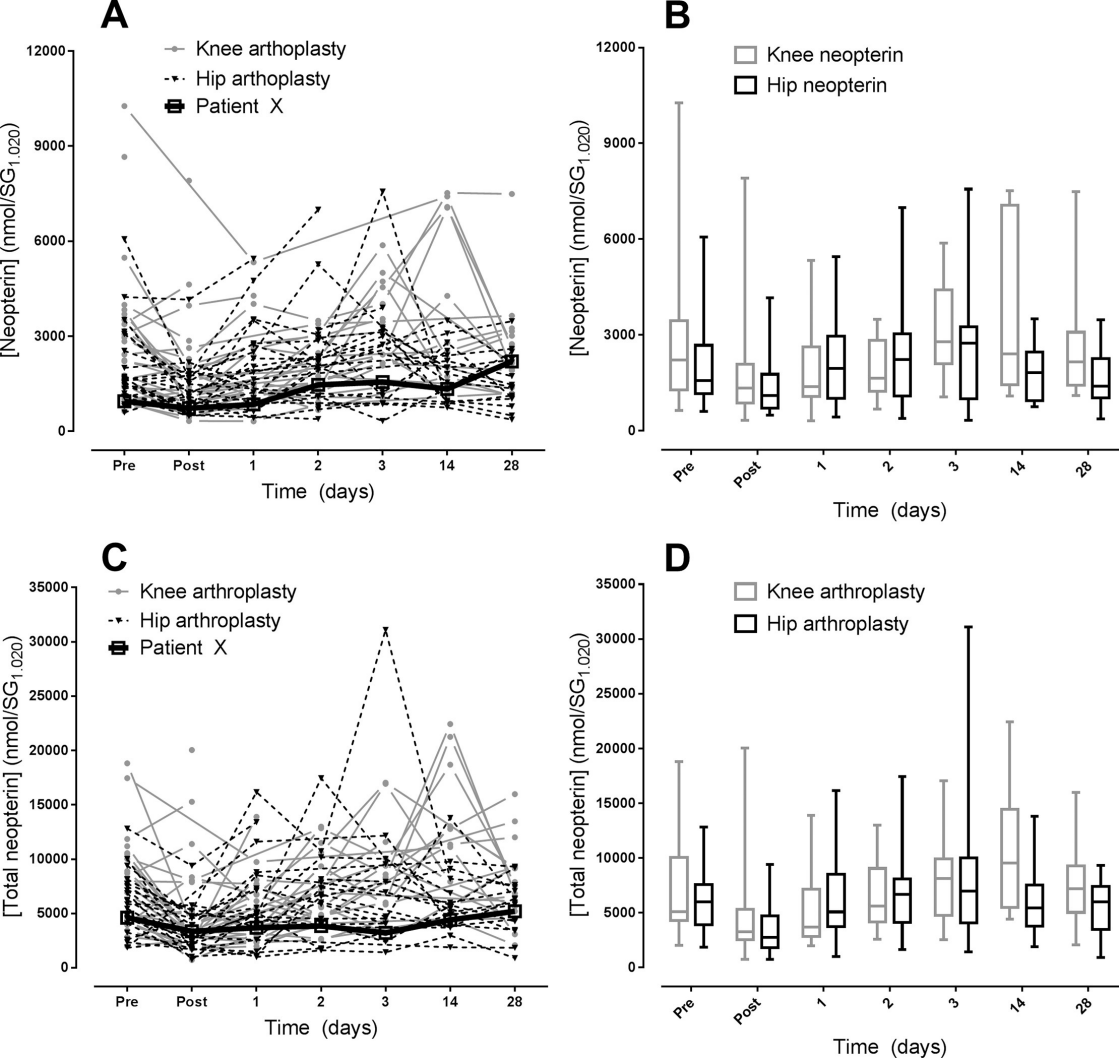
Individual plots (A and C) and box and whisker plots (B and D) for urinary neopterin (A and B) and total neopterin (C and D) to demonstrate inter- and intra-group variation. Each line represents a single participant. Patient X was a knee arthroplasty patient who was highly active before and shortly after surgery. Box and whisker plots depict medians, upper and lower quartiles and minimum and maximal values.

The biomarkers profiles of individual participants are shown as single lines (Fig 3A and 3C), with group data also being graphed as box and whisker plots to better describe the intra- and intergroup variation (Fig 3B and 3D). The individual data shows that the knee arthroplasty cohort contains multiple participants who had high urinary neopterin and total neopterin values at day 14 post surgery. There was also large variation between patients of the same group, with one highly physically activate patient (patient X) showing a very limited change in urinary neopterin and total neopterin values following surgery. Patient X reported travelling 250 km per week on a bicycle up to one month pre-surgery and begun minor bicycling 14 days following surgery. By 28 days post-surgery, patient X reported 100 km/week. The variation in individual patient values did not correlate with any other variables recorded (pain, medication, anesthetic usage).

**Fig 4:**
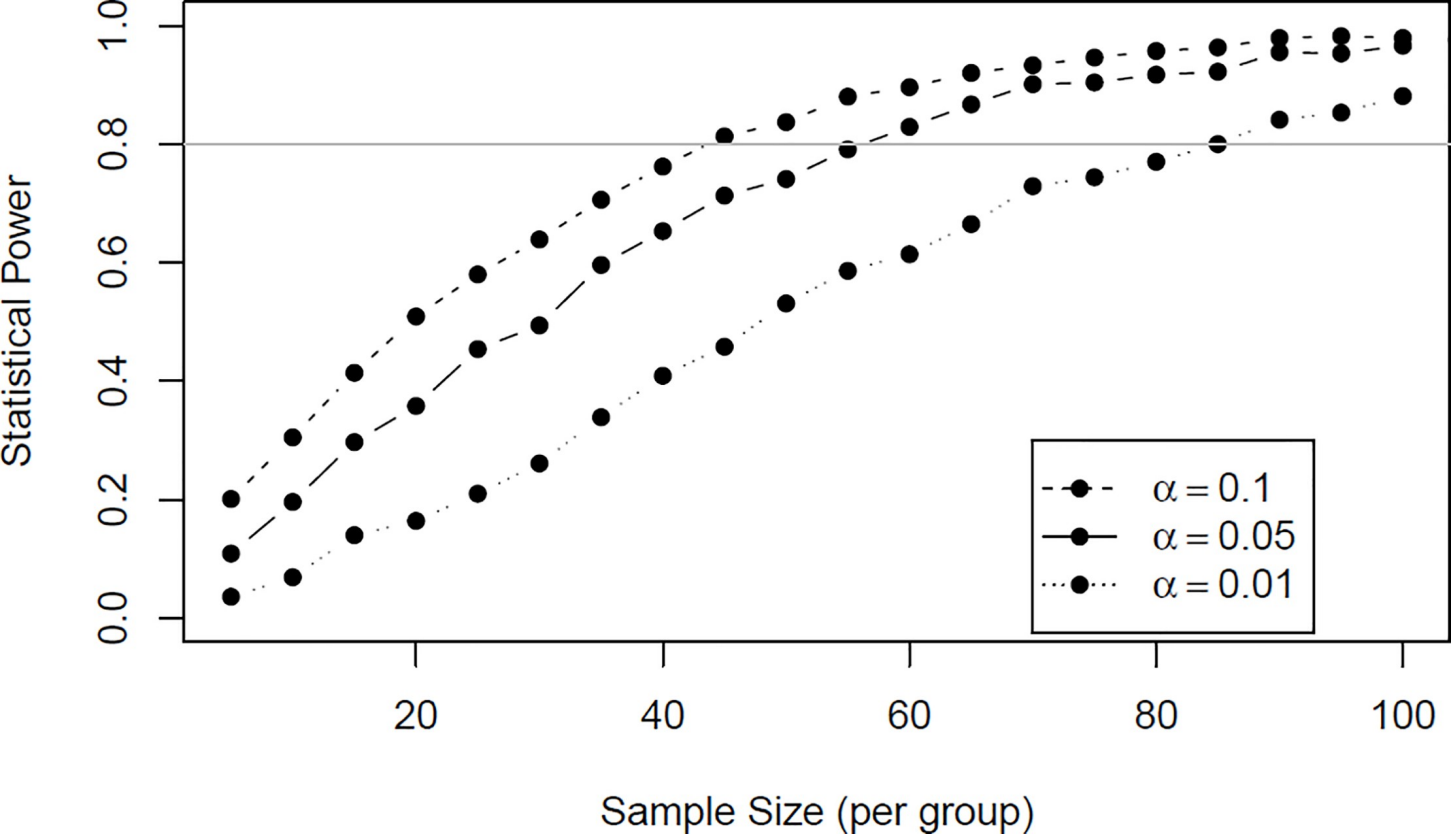
Post-hoc power analysis shows that approximately 55 participants per group with full observations would be required to demonstrate a difference at significance level of α = 0.05 with 0.80 power for day 14 post-surgery.

A post-hoc power analysis was conducted to determine the required cohort sizes to detect a statistically significant (α = 0.05) difference between the neopterin levels following knee and hip arthroplasty at day 14 (Fig 4). With 0.80 power this would require approximately 55 participants in each group with full observations. For α = 0.1 approximately 97 participants would be required.

## Discussion

Neopterin and total neopterin have been shown to be informative biomarkers in measuring grouped and individual data for oxidative stress and immune cell activation in participants exposed to physical trauma [15,25,28,29]. In this study we have shown that urinary total neopterin values were significantly higher for knee arthroplasty patients versus hip arthroplasty patients before and after surgery. Urinary neopterin is also elevated in knee patients versus hip arthroplasty patients, but not statistically significantly.

Elevated levels of urinary total neopterin supports our first hypothesis and indicates that hip arthroplasty results in higher levels of inflammation that knee arthroplasty. (Fig 1). Pre-surgical mean levels of urinary neopterin and total neopterin are higher in the knee arthroplasty group. This observation may be due to a greater inflammatory component when the knee is affected by osteoarthritis than the hip. Furthermore, this has been seen in a previous study where urinary neopterin and total neopterin were elevated before surgery versus healthy controls [25]. The progression of osteoarthritis has also been associated with the level of measured inflammation using multiple biomarkers [30]. Neopterin may provide more reliable information regarding inflammatory status of a patient by being more sensitive than CRP or IL-6, which have not been able to distinguish between knee and hip osteoarthritis or subsequent arthroplasty surgery [3–5].

There was a significant post-surgical decrease in both neopterin and total neopterin which has been reported with the use of anesthesia [25,31]. In this study there was a highly significant (p<0.001) rebound increase in urinary neopterin and total neopterin up to 28 days following this post-surgical dip, peaking at day 3 and 14 for hips and knees respectively. This has been seen previously in urinary neopterin levels following knee arthroplasty monitored for three days post-surgery [25], as well as, serum CRP levels [5]. The mean levels of neopterin and total neopterin were considerably higher in knee arthroplasty patients than hips at day 14. Additionally, by day 14 mean neopterin and total neopterin levels for hip arthroplasty had returned to pre-surgical levels, whereas, the levels for knee arthroplasty remained slightly elevated and did not return to pre-surgical values until day 28, suggesting that knee arthroplasty may result in more oxidative stress and inflammation. As the grouped difference between knee and hip arthroplasty for neopterin and total neopterin at day 14 was not statistically significant a post-hoc power analysis was conducted and demonstrated that 55 participants would be required in each group to prove the observation to a level of p<0.05, assuming the observed mean difference was a real effect (Fig 4). Combined, these results suggest that knee arthroplasty not only led to more oxidative stress and inflammation, but also a slower inflammatory resolution compared to hip arthroplasty. This observation is further supported by clinical studies that have investigated long term outcomes and hormone levels [1,3,6].

The ratio between total neopterin (or 7,8-dihydroneopterin) versus neopterin has been used clinically to assess the dynamics between oxidative stress (neopterin) and inflammation (total neopterin) [17,23,25]. While this study did not see any difference between the TNP/NP ratios of knee and hip arthroplasty patients (Fig 2), it does show that the respective peak ratios of 4.06 and 3.85for knee and hip arthroplasty are larger than the TNP/NP ratio of 2.51 previously seen in healthy controls [25]. This indicates that there is a greater production of 7,8-dihydroneopterin relative to neopterin, which suggests there is relatively more monocyte and macrophage cell activation than oxidative stress being generated by the immune system [17].

Furthermore, this result indicates that the dynamics between oxidative stress and monocyte/macrophage activation for hip and knee arthroplasty surgery are equivalent, irrespective of knee arthroplasty generating overall higher levels of oxidative stress and inflammation. The equivalence of the TNP/NP ratio here suggests that even with the varying levels of oxidative stress and inflammation seen between knee and hip arthroplasty patients, the body’s response in upregulation of oxidative stress and inflammatory pathways is equal.

Increased inflammation with limited oxidative stress is also more characteristic of tissue destruction and remodeling which would be expected following a physical trauma without infection, such as arthroplasty surgery [32,33].

The individual data shows substantial variation between patients in, and between, the two groups (Fig 3). Attempts were made to generate a prediction model in which pre-surgical values could be used to estimate day 14 and 28 levels. However this was unsuccessful, indicating that neopterin and total neopterin are unlikely to be useful predictive markers of recovery. This is likely to be due to the large variation seen from patient to patient and inherent variation from surgery to surgery. High levels of inter-participant variation in neopterin and total neopterin have been seen in exercise trauma studies [24,34]. Most of the participants in this study were relatively physically inactive due to being elderly and debilitated by osteoarthritis. However, one knee arthroplasty patient (Patient X, Fig 3) was highly active and biked 250 km per week until one month prior to the surgery. This patients urinary neopterin and total neopterin levels were extremely low, relative to other participants, but began to rise at days 14 and 28 which coincide with the increased cycling the patient was doing (100 km/week by day 28). An individual’s level of fitness, physical activity and conditioning has been shown to greatly impact the immune system and subsequent neopterin levels following trauma [11,35,36]. In juxtaposition, the effectiveness of pre-surgical exercise regimes has not been shown to have a significant effect on post-operative function or recovery, however these studies did not measure or focus on inflammatory biomarkers [37,38]. While a meaningful conclusion cannot be ascertained from a single participant, we speculate that the limited response to knee arthroplasty seen in patient X may be due to a high degree of physical fitness and conditioning. Further studies to investigate this potential effect would involve patients with a pre-determined and measurable level of fitness and activity.

7,8-Dihydroneopterin and its oxidation product neopterin are intimately tied to activity of the immune system and levels of oxidative stress [10,20,39]. As such these biomarkers have been extensively studied in infection and trauma [20,40–42], with infection showing higher levels of neopterin production than significant physical trauma [43]. Moreover, neopterin has been shown to have some prognostic capabilities as an early warning biomarker for infection in critically ill patients [40,44]. While none of the patients in this study were afflicted by post-surgical infection, it seems possible, if not likely, that if infection were to occur neopterin and total neopterin levels would spike far higher than what would otherwise be expected from the surgical trauma alone. The lack of complications seen throughout the study is a testament to the skill and care of the surgical and post-operative teams. Having the ability for clinicians to quickly and conveniently analyses a patient’s level of immune system activation may be a highly valuable tool in situations where rapid determination of immune functioning is required for decision making, such as the development of infection or in limiting a secondary immune hit following trauma which has been shown to reduce patient outcomes [45].

## Conclusion

In this study we have shown that both knee and hip arthroplasty surgeries cause an increased level of oxidative stress and inflammation following the anesthetic mediated post-surgical dip in urinary neopterin and total neopterin. We have confirmed our hypothesis that this increase is larger for knee arthroplasty versus what was seen from hip arthroplasty surgery. This indicates that osteoarthritis in the knee and the knee arthroplasty procedure cause a higher level of immune activation, likely due to increased complexity and loading on the joint, as well as, increased physical trauma from the surgery. Also we have confirmed that urinary neopterin and total neopterin levels fall faster following a hip arthroplasty, indicating a faster inflammatory recovery. The measurement of urinary neopterin and total neopterin provide a non-invasive measure of patient’s levels of oxidative stress and inflammation in response to surgical procedures and may be valuable in improving the clinical outcomes in these patients.
